# Sulfated Glycans and Related Digestive Enzymes in the Zika Virus Infectivity: Potential Mechanisms of Virus-Host Interaction and Perspectives in Drug Discovery

**DOI:** 10.1155/2017/4894598

**Published:** 2017-01-19

**Authors:** Vitor H. Pomin

**Affiliations:** ^1^Program of Glycobiology, Institute of Medical Biochemistry Leopoldo de Meis, Federal University of Rio de Janeiro, 21941-590 Rio de Janeiro, RJ, Brazil; ^2^University Hospital Clementino Fraga Filho, Federal University of Rio de Janeiro, 21941-913 Rio de Janeiro, RJ, Brazil

## Abstract

As broadly reported, there is an ongoing Zika virus (ZIKV) outbreak in countries of Latin America. Recent findings have demonstrated that ZIKV causes severe defects on the neural development in fetuses in utero and newborns. Very little is known about the molecular mechanisms involved in the ZIKV infectivity. Potential therapeutic agents are also under investigation. In this report, the possible mechanisms of action played by glycosaminoglycans (GAGs) displayed at the surface proteoglycans of host cells, and likely in charge of interactions with surface proteins of the ZIKV, are highlighted. As is common for the most viruses, these sulfated glycans serve as receptors for virus attachment onto the host cells and consequential entry during infection. The applications of (1) exogenous sulfated glycans of different origins and chemical structures capable of competing with the virus attachment receptors (supposedly GAGs) and (2) GAG-degrading enzymes able to digest the virus attachment receptors on the cells may be therapeutically beneficial as anti-ZIKV. This communication attempts, therefore, to offer some guidance for the future research programs aimed to unveil the molecular mechanisms underlying the ZIKV infectivity and to develop therapeutics capable of decreasing the devastating consequences caused by ZIKV outbreak in the Americas.

## 1. Introduction: The Consequences of the Zika Outbreak in the Americas!

As largely broadcasted worldwide, there is currently a Zika virus (ZIKV) infection outbreak in Latin America, including Rio de Janeiro, Brazil [1–5], which has held the Summer Olympics of 2016. The ZIKV infection outbreak has started at the beginning of 2015. Brazil was the first country of Latin America to officially report an outbreak related to ZIKV to the international authorities [6]. Speculations exist concerning how and when the ZIKV has endemically entered into Brazil. It was initially believed that ZIKV had gained access into the country likely through infected Africans coming to attend the 2014 World Cup. The association of the two events, the 2014 World Cup and the 2015-2016 ZIKV epidemic in Brazil, is quite reasonable. Nowadays, it is generally believed that the virus had gained access into the country prior to the World Cup. Nonetheless, no real proofs have been found or postulated so far about the virus origin, about the starting time window of infection, and about the ways of propagation during the initial period of the outbreak. The current problem in public health caused by ZIKV [7] in Brazil has been indirectly aggravated by ongoing internal political and economic conflicts.

The clinical signs and/or symptoms of adults suffering from ZIKV-caused fever are not that serious, and health in the patients can be recovered by traditional medicinal methods in a relative easy way. However, if pregnant women and infants are found among the infected population, the consequences can be very tragic and, at a certain point, clinically unmanageable [8, 9]. It has been recently reported by numerous media sources that ZIKV infection is a tremendous risk to pregnant women or those planning pregnancy [10, 11]. This relies on the fact that ZIKV can seriously damage and compromise the brain development of the fetus in utero. The terrible consequences of this neural damage, named congenital microcephaly, are clinically irreversible and are caused by decreased neuronal production as a consequence of proliferative defects and/or deaths of the cortical progenitor cells [12]. Primary microcephaly occurring in fetuses or newborns leads to a severe set of neural abnormalities. It includes malformation of the brain morphology and physiology as well as anatomic defects of the head [6]. The reduced circumference of the head in newborn babies committed with microcephaly is a clear visual sign of the devastating consequence of the ZIKV infection and the epidemic in the Americas. The malformation of the head and the brain is originated by defects on the neural development of fetuses during the time of pregnancy. Primary microcephaly associated with brain impairments on the born and unborn babies like visual, hearing, and cognitive dysfunctions and motor disabilities were all consequences caused by ZIKV [13]. This is because ZIKV can cause additional fetal abnormalities in organs other than just brain like eyes, ears, and limbs. Some of the neural defects caused by ZIKV are quite similar to those already described for the* Guillain-Barré* syndrome that occurs in adults [14].

In the beginning of 2016, the US Centers for Disease Control and Prevention issued travel guidance and warnings regarding affected countries of Latin America, including the use of enhanced precautions, and advised pregnant women to consider postponing their travels [15, 16]. A similar warning notice has been issued by the World Health Organization [17]. The chair of this international entity has visited Brazil in order to pursue a short-term evaluation together with the local health authorities on the alarming and devastating consequences of the ZIKV infectivity in pregnant patients carrying babies diagnosed with microcephaly and in infant children committed with the ZIKV-caused birth defects. Warnings have been also sent to tourists willing to visit the country in 2016 during the periods of international events like Carnival and the Summer Olympic Games [18].

## 2. General Characteristics of the ZIKV and Its Infectivity

ZIKV is a member of the virus family Flaviviridae, genus* Flavivirus* [19]. Like other flaviviruses, ZIKV is an enveloped icosahedral virus composed of a nonsegmented, single-stranded, positive-sense RNA as genetic material [20]. This explains why the pathology caused by ZIKV shares common features with diseases caused by other flaviviruses such as dengue, yellow fever, Japanese encephalitis, and West Nile viruses [21]. Like most flaviviruses, ZIKV is spread by daytime-active* Aedes* mosquitoes, such as* A. aegypti* and* A. albopictus* [14]. The virus name derives from the Zika Forest of Uganda, where the virus was first isolated in 1947 [21]. One of the biggest reasons for the increased rates of infection and propagation of ZIKV in the countries of Central and South America is the fact that the transmissible vectors are ubiquitously spread in the continent, as a consequence of the tropical climate.

ZIKV has the capacity of infecting not only dendritic cells, dermal fibroblasts, and epidermal keratinocytes located close to spots where the mosquito has bitten [22] but also nervous cells [23], neural stem cells [24], and brain organoids [24]. The capacity of ZIKV to attack and damage developing and mature elements of the nervous tissue on fetuses in utero was deduced by clinical facts of observing the primary microcephaly occurring in newborns from pregnant women who had historically suffered from ZIKV fever during their pregnancies. Considering the year of publication of Bell and associates (1971) [23], the evidence of the potential danger to the nervous system caused by ZIKV is not a recent scientific finding. The novel finding recently reported was the association of the ZIKV infectivity with the severe brain damage in fetal development. This association had not been anticipated from the publication of Bell and coworkers made 45 years ago [23].

Unfortunately, this association was made through a sad way which was the observation a great number of newborns committed with severe birth neural defects coincidently appearing in a very short period of time in just certain cities of Brazil. Recently, investigations have indicated additional ways of transmission that could have contributed to the growing rates of the ZIKV epidemic in the countries of Latin America. Besides the commonly known vector-borne and mother-to-child (vertical) transmission, ZIKV can also be spread via sexual contact [25–27] and blood transfusion [28].

Nowadays, the relationship between the occurrence of microcephaly in newborns and ZIKV has been established [29]. In this recently published review paper, the authors have highlighted the original experimental evidences that have strengthened the potential link between ZIKV and the high incidence of microcephaly in newborn babies.

## 3. The Possible Role of Glycosaminoglycans in the ZIKV-Host Interaction

As is common to the series of enveloped RNA-composed virus including the mosquito-borne flaviviruses, glycosaminoglycans (GAGs) displayed at the surface proteoglycans of host cells must form a first-moment molecular complex with protein embedded on the envelope viral particle in order to trigger the viral infection [30–33]. This GAG-dependent molecular mechanism occurs not only in viruses belonging to the Flaviviridae family but also in members of other families. Examples are herpes simplex virus (HSV) [34], cytomegalovirus [35], both belonging to the Herpesviridae family, and papillomavirus (Papillomaviridae family) [36]. Hence, it is a common mechanism of action for most viral infections. In most of these cases, GAGs are considered as more attachment factors than coreceptors per se because they are not “directly” responsible for the entry of viruses into the host cells but help the function of the primary virus receptors on cells or viral particles to concentrate on surface of host cells prior to the virus invasion.

**Figure 1:**
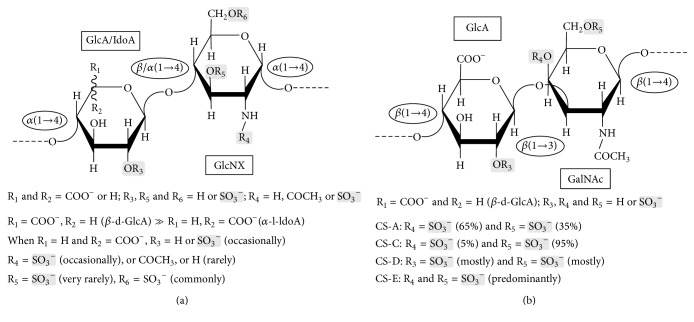
Structural representation of (a) heparan sulfate (HS) and (b) chondroitin sulfate (CS) and subtypes. (a) The repeating disaccharide unit of HS is composed of alternating 4-linked uronic acid and 4-linked *α*-glucosamine (GlcNX) units. The uronic acid can be either *β*-d-glucuronic acid (GlcA) as its major component or *α*-l-glucuronic acid (IdoA). Regarding possible substitutions, the GlcNX units are frequently* N*-acetylated (GlcNAc), occasionally N-sulfated (GlcNS), and rarely* N-*free (just the amino NH_2 _group, as GlcN).* O*-sulfation can occur at both units of HS. While 2-*O*-sulfation occurs occasionally at the IdoA, 6-*O*-sulfation is common at the GlcNS units. 3-*O*-sulfation may also occur at the occasional GlcNS6S unit but very rarely in terms of amounts. (b) The repeating disaccharide unit of CS is composed of alternating 4-linked*β*-d-glucuronic acid (GlcA) and 3-linked *β*-d-galactosamine (GalNAc) units. The CS subtypes differ according to sulfation patterns as follows: CS-A is mostly 4-*O*-sulfated at the GalNAc units, CS-C is predominantly 6-*O*-sulfated at the GalNAc units, CD-D is mostly 2-*O*-sulfated at the GlcA units and 6-*O*-sulfated at the GalNAc units, and CS-E is mostly 4,6-*O*-disulfated at the GalNAc units. In both panels, the glycosidic bonds are indicated inside the ellipses, whereas monosaccharide types are indicated inside the rectangles. For fast notation of the sulfation patterns, sulfation sites are highlighted by light-grey shadowed rectangles.

During infection of various viruses, the GAG types, heparan sulfate (HS) (Figure 1(a)) and chondroitin sulfate (CS) (Figure 1(b)), modulate virus attachment and binding onto the host cells. In a posterior moment, these sulfated glycans can also assist the virus entry into the host cells. For example, HS is the host cell surface molecule targeted by the envelope protein of dengue virus during the initial steps of its infectivity [37]. CS-E (Figure 1(b)) is known to be the binding motif in CS proteoglycans of the host cell for the development of the Japanese encephalitis viral infection [38]. While CS may work as attachment receptor in viral infections occurring at the nervous cells [37], likely as a result for being the most abundant functional GAG of the nervous system [39, 40], HS serves as viral attachment receptor in infections occurring in skin-related cells [41], given its high abundance in this tissue [42]. The HS-dependent mechanism is widely studied for the case of the human immunodeficiency virus (HIV) [43–50]. In those cases, HS plays a key role in binding to the envelope-embedded glycoprotein 120 (gp120) during the HIV adhesion onto the host CD4^+^ T cells [43, 46, 48–50]. The HS-gp120 complex is aimed as possible target for multiple potential antiviral candidates against HIV [49, 50]. The glycoprotein 41 (gp41) seems to also present some additional binding properties to the host cell HS [45].

In analogy to the above-illustrated case of HIV extensively studied by independent experts from different laboratories of the world and as commonly observed during infections caused by many other virus types including those belonging to the Flaviviridae family, the formation of the intermolecular complex of host cell GAGs with surface proteins of ZIKV is very likely to occur. Following the consensus, in case this flavivirus infects skin-related cells such as dendritic cells, dermal fibroblasts, and epidermal keratinocytes nearby the spots of the mosquito bite, HS (Figure 1(a)) is the most probable GAG type to work as functional attachment receptor for the ZIKV. In case this flavivirus infects nervous cells such as neurons or neural stem cells, CS (Figure 1(b)) is likely the most probable GAG type to function as ZIKV attachment receptor. The premise of HS playing a role in skin cells and CS in neurons during the ZIKV infectivity is based solely on the abundance and specific occurrence of these GAG types in these particular tissues. Although scientific results regarding the GAG-dependent mechanisms in ZIKV infectivity are still virtually inexistent up to this moment, primarily because ZIKV epidemic is quite recent in time, the speculations raised regarding the GAG-dependent mechanisms of ZIKV-host interaction are fairly reasonable in light of considering the general mechanisms of infectivity of most viruses. These speculations must be broadly shared with the scientific community involved in research on ZIKV.

## 4. Potential Sulfated Glycan-Related Therapeutic Strategies against ZIKV

Assuming that the intermolecular complex made by host cell GAGs and viral surface proteins must be assembled in order to trigger the viral infectivity, inhibitors and competitors of the host cell GAGs, and even enzymes able to cleave these surface GAGs of host cells, may be medically explored in order to decrease the levels of the virus infectivity. This therapeutic strategy has been reported in numerous publications regarding various viruses. The goal of obtaining or designing potential sulfated glycans that could be exogenously applied as potential therapeutics against viral infections is ultimately quite relevant to medicine [51].

For instance, sulfated glycans of various origins and distinct structures have been identified and investigated against multiple viral infections, especially those caused by enveloped viruses such as HSV, cytomegalovirus, vesicular stomatitis virus, and HIV [52]. Classes of these potential sulfated glycans mostly investigated against the cases of HIV are (1) isolated GAGs like CS [53], dermatan sulfate [54], HS [50], heparin [55, 56], HS conjugated with CD4 [57], and the structurally uncommon GAGs isolated from marine organisms [58] such as the holothurian fucosylated CS [59] and the ascidian dermatan sulfates of unique sulfation patterns [60]; (2) marine-derived sulfated glycans such carrageenan [61, 62], agarans [63], sulfated galactans [64, 65], and sulfated fucans/fucoidans [66]; and (3) synthetic glycans such as GAG analogs and/or structurally modified GAGs [67–70], commercially available dextran sulfates [56, 66, 71, 72], and pentosan polysulfate [73], among other examples.

Sulfated glycans, employed as competitors to the host cell GAGs, are not the exclusive potential therapeutics available for investigation in medicine. Enzymes capable of degrading cell GAG molecules such as heparinases, heparitinases, hyaluronidase, and chondroitinases are additional compounds available and potentially endowed with beneficial properties against viral infections. These digestive enzymes are capable of cleaving the supposed GAG attachment receptors from the host cells, therefore reducing the levels of infectivity from the viruses. The loss of activity of the GAG attachment receptor, the consequential virus binding, and the reduced incidence of infectivity have been successfully reported for the case of Chikungunya virus, a mosquito-transmissible alphavirus which is also threatening the public health in territories of the Latin America like ZIKV [30]. As for Chikungunya, the medical exploration of GAG-digestive enzymes is likely a path in drug development for the case of ZIKV.

Hence, the medical effects of (1) sulfated glycans of different sources and chemical structures and (2) GAG-degrading enzymes should be clinically investigated against ZIKV. This is particularly important and urgent in order to avoid the harmful consequences onto the nervous system of fetuses in pregnant women and newborns. The sulfated glycans and related enzymes can be, therefore, considered as potential therapeutics readily available for use in order to decrease and control the devastating consequences caused by ZIKV outbreak in Latin America. Specific goals for future research programs concerning ZIKV infections could be followed by researchers in order to speed the comprehension about the molecular mechanisms underlying the ZIKV infectivity and to develop adequate therapeutics able to enhance the combat against the ZIKV outbreak and its harmful impact on the public health. Some of these research goals are described in the next section.

## 5. Conclusions, Perspectives, Goals for Future Research Programs, and Challenges

A ZIKV infection outbreak is occurring in countries of Latin America. The large spread is a consequence of the abundance of the mosquito vectors responsible for transmitting and propagating the ZIKV. Although the symptoms are usually mild in adults, the consequences can be very tragic in case pregnant women and infant newborns are infected with this arbovirus. This relies on the fact that ZIKV can damage the neural system during its development in fetuses in utero and in newborn babies. Microcephaly, brain impairments, and compromised visual, hearing, cognitive, and motor functions are all severe consequences caused by ZIKV. ZIKV is a flavivirus able to infect not only skin-related cells located nearby areas where the mosquito has bitten but also cells of the nervous system.

As commonly observed in infections of the most viruses, including flaviviruses, an intermolecular complex made by GAGs at the surface proteoglycans of host cell and GAG-binding proteins of the virus surface must be formed in order to initiate the viral infectivity. While HS serves as virus attachment receptor in skin-associated cells, CS serves in the nervous cells. Both GAG types play key roles in virus attachment onto the host cells and, sometimes, in consequential virus entry into the cells. These GAGs can be aimed as target molecules in many research programs concerning development of antiviral agents. GAG analogs and/or inhibitors can be exogenously applied into infected systems as potential competitors against the functional GAGs required to the progress of the viral infectivity. Among these competitors, sulfated glycans of different origins and of various chemical structures have been largely investigated by independent laboratories of the world. GAG-degrading enzymes able to remove the GAG-based virus attachment receptors are also promising molecular alternatives available in medicine for application against viruses. They can be used in fights against virus types that depend on host cell GAG attachment receptor to the progress of their infectivities. ZIKV is likely a GAG-dependent virus like many other members of the Flaviviridae family.

We hope to see the decrease of the incidence rates of the ZIKV epidemic in the Americas in the next months, not only through the principal method of disease control which is the direct elimination of the mosquito vectors and their proliferative spots but also through additional clinical methods such the employment of therapeutically active molecules able to act on the infected system. Sulfated glycans and GAG-degrading enzymes are among these compounds available for clinical experimentation and for further clinical trials in order to develop new and effective anti-ZIKV agents. Unlike vector control which focuses on prevention, the molecular methods offer a possible treatment to infected patients. This is particularly important for pregnant patients infected with ZIKV in order to avoid systematic virus spread and consequential damage of the fetal neurons.

However, the design and development of the strategies suggested here will not be trivial for implementation. A series of precautions and investigations must be followed prior to having the sulfated glycans and related digestive enzymes capable of benefitting the community. Firstly, tests concerning the possible adverse side effects of sulfated glycans, or GAG-degrading enzymes, must be performed in order to prevent unintentional harm to patients since the interference with the physiological GAG functions in animals and humans may lead to bad consequences to health. This is because GAGs are essential molecules ubiquitously displayed at the surface of all cell and tissues. The strategies designed to block their regular functions with a pharmacological purpose must be well-controlled and directed almost exclusively to the ZIKV-related infectivity. Otherwise the other biological functions of GAGs will be compromised. In addition to that, trials on laboratory animals must be implemented in order to evaluate the efficiency, efficacy, bioavailability, and dose-response of these suggested therapeutic strategies. Secondly, bioavailability of the possible carbohydrate-related therapeutics aimed to be used against ZIKV infectivity must be deeply analyzed. The difficulties in developing antiviral agents capable of adequately penetrating the central nervous system are largely known. Another point to be investigated is the feasibility of these sulfated glycans and related enzymes capable of crossing through the placenta during pregnancy, so that they can be functional to protect the fetuses in uterus. Thirdly, another challenge of this moment would be the research on the potential medical contributions of marine medicinal compounds as recently suggested in the research program entitled* Marine Medicinal Glycomics* [58].

GAGs are essential for attachment and infectivity of many viruses and other virus-related events. It has been postulated that attenuated yellow fever virus (YFV) strain 17D vaccine [74] and both cell adapted YFV and dengue virus type 2 (DENV 2) New Guinea C strain need GAGs, especially HS for proper function [75]. Additionally, tick-borne encephalitis was also shown to only bind GAGs (HS) when cell culture adapted [76]. In order to enable its virulence, ZIKV can either bind naturally to GAGs (HS or CS) during infectivity like Eastern equine encephalitis virus (EEEV) [76, 77], or bind artificially to GAGs like Sindbis virus (SINV) [77], or request cell adaptation like neuroadapted SINV and EEEV [77]. There are also other RNA viruses that do not depend on GAGs but can still be neurovirulent. These different aspects of GAG-dependent (or independent) mechanisms of infectivity remain unclear for ZIKV.

Although no scientific data have been presented in this communication, the ideas described herein concern key topics to be investigated in the future research programs related to ZIKV infectivity. These ideas must be shared with the community, especially with those investigators aiming to perform future research on carbohydrates, related enzymes, and their functions in viral infectivity of ZIKV. Specific goals for future research programs involved in the battle against ZIKV infection could be the series of analysis on the following aspects: (1) the carbohydrate-related molecular mechanisms involved during the infectivity, notably GAGs involved in the initial event of virus-host cell interaction; (2) the role of HS as the major GAG component on proteoglycans of infected skin-related cells; (3) the role of CS as the major GAG type of the nervous system during the progress of congenital microcephaly and in cases of ZIKV infections on neurons; (4) the possible anti-ZIKV response of the various sulfated glycans assayed through in vitro and in vivo models that could simulate the infected conditions; (5) the medical mechanisms of the potential sulfated glycans against ZIKV as reported to other flavivirus-caused infections [78, 79]; (6) the possibility of undertaking clinical trials for the future medical use of exogenous sulfated glycans capable of competing with the GAG attachment receptors supposedly required for ZIKV infectivity; (7) the medical evaluation of GAG-degrading enzymes as potential therapeutic agents against the progress of the ZIKV infection, considering the surface HSs and CSs (Figure 1) of host cells as their common substrates as previously reported to other viral diseases; (8) the possibility of performing clinical trials for the consequent medical application of GAG-digestive enzymes able to remove the host cell attachment receptors required to the progress of the ZIKV infectivity; (9) the mechanisms of GAG (HS/CS)-dependent infectivity of ZIKV as described for other RNA viruses (a) via natural binding such as EEEV, (b) via artificial binding such as SINV, and (c) via cell adaptation for neurovirulence as described for cell adapted SINV and EEEV [76], and (10) the capacity of the various sulfated glycans and related degrading enzymes in trespassing through the blood-brain barrier and the placenta in pregnant women. The ninth aspect is of great importance. Since there are few neurovirulent RNA-constituted viruses that do not necessarily bind and need GAGs for infectivity, it would be a big surprise to see this phenomenon also occurring on ZIKV infectivity. If GAG-independent mechanisms were discovered for ZIKV infectivity, most of the goals above suggested for the future research programs of ZIKV would be worthless. Hence, the solution to this ninth aspect is of high-priority. Regardless of the outcome on this matter, the biological role of sulfated GAGs in the ZIKV infectivity must be targeted in the next generations of studies concerning the molecular mechanisms and possible therapeutic agents involved in ZIKV whose outbreak is threatening the pregnant and child population of the countries of Latin America in a large epidemic scale.
